# Dual Roles of PTSA in Electrical Conductivity of PEDOT:PTSA with Large Seebeck Coefficient

**DOI:** 10.3390/ma18030619

**Published:** 2025-01-29

**Authors:** Hideki Arimatsu, Yuki Osada, Ryo Takagi, Yosuke Ohira, Tomoki Hijikata, Takuya Fujima

**Affiliations:** Department of Mechanical Engineering, Tokyo City University, Tokyo 158-8557, Japan; mkrnsld1226@outlook.jp (Y.O.); ryo_rascal@icloud.com (R.T.); yousuke.soccer12@gmail.com (Y.O.); hijitomo1999@gmail.com (T.H.); tfujima@tcu.ac.jp (T.F.)

**Keywords:** PEDOT, PTSA, organic thermoelectric materials, thermoelectric properties

## Abstract

The electrical conduction mechanism of PEDOT:PTSA thermoelectric conversion material supported on PET fiber was investigated with varying PTSA concentrations. Raman analysis revealed that an increasing PTSA concentration promoted transformation from a benzoid to a quinoid structure in PEDOT chains, reaching saturation in higher concentrations. All samples exhibited p-type behavior, with Seebeck coefficients ranging from 0.9 to 2.7 mV/K. The temperature dependence of electrical conductivity showed that conductivity and activation energy exhibited extreme values with increasing PTSA concentration, correlating with the saturation of quinoid structure transformation. This behavior suggests that PTSA serves dual roles: at lower concentrations, it enhances electrical conductivity through chemical doping, increasing carrier concentration and mobility via quinoid structure formation; at higher concentrations, excess PTSA induces carrier scattering without contributing to chemical doping, thereby reducing conductivity. These findings indicate that the thermoelectric properties of PEDOT:PTSA on PET fiber are governed by the balance between chemical doping effects and carrier scattering mechanisms, which are both influenced by PTSA concentration.

## 1. Introduction

As Internet of Things (IoT) devices have become more prevalent, extensive research has been conducted on energy-harvesting technology for independent power sources and sensors [1,2,3]. One such energy-harvesting method, thermoelectric power generation, transforms temperature differentials into electrical energy. Given its ability to generate electricity from heat produced by the human body, thermoelectric power generation is currently under investigation and development for practical applications [4,5].

The dimensionless figure of merit *ZT* = *S*^2^*σT*/*κ* is used to assess the effectiveness of thermoelectric conversion materials, where *S* is the Seebeck coefficient, *σ* is the electrical conductivity, *κ* is the thermal conductivity, and *T* is the temperature [6]. Higher *ZT* values correspond to increased thermoelectric power generation efficiency, necessitating materials with enhanced Seebeck coefficients and electrical conductivity and reduced thermal conductivity. However, a trade-off exists between the Seebeck coefficient and the electrical conductivity in metals and semiconductors.

Thermoelectric conversion materials are classified as p-type or n-type based on their positive or negative Seebeck coefficients. Thermoelectric power generation devices usually consist of p- and n-type materials connected electrically in series and thermally in parallel [7]. Developing both p- and n-type thermoelectric materials within the same material system is demanded for practical power generation to prevent fracture of the generation unit due to thermal distortion [8,9].

The development of flexible thermoelectric conversion materials is crucial for their integration into wearable devices, which require bendability. Organic thermoelectric conversion materials show promise in this area because of their low thermal conductivity, cost-effectiveness, and high flexibility [10,11,12]. Among these, a conductive polymer, PEDOT:PSS, which consists of Poly3,4-ethylenedioxythiophene (PEDOT) chemically doped with polystyrene sulfonate (PSS), has attracted significant attention for practical applications in organic thermoelectric conversion. This is primarily due to its high electrical conductivity and chemical stability [13,14]. Although PEDOT:PSS has been noted to have a high *ZT* among organic thermoelectric conversion materials [15], its performance still lags behind that of Bi_2_Te_3_, which is currently used in practical applications.

Research efforts to enhance the thermoelectric properties of PEDOT:PSS have primarily focused on two key parameters: the Seebeck coefficient and electrical conductivity. The enhancement of the Seebeck coefficient has been approached through the incorporation of various composite materials. Notable examples include the integration of Bi_2_Te_3_ and CNT, which utilize the energy filtering effect to modify the thermoelectric properties [16,17,18]. However, these composite materials typically exhibit positive Seebeck coefficients, highlighting an ongoing challenge in developing n-type PEDOT-based materials for practical applications.

In parallel, strategies to improve electrical conductivity have developed along two main paths. The first approach involves adding high-boiling-point solvents [19,20,21], while the second explores using alternative dopants to replace PSS [22,23]. A significant challenge in the latter approach is that removing PSS substantially compromises film formability. To address this limitation, researchers have developed various solutions, including the use of surface-modified substrates [24] and the implementation of macrostructural separation layers [25].

First-principles calculations have predicted that PEDOT:PTSA possesses the potential for large Seebeck coefficients due to the formation of sharp impurity levels at the valence band edge [26]. An experimental study has demonstrated that PEDOT:PTSA supported on polyethylene terephthalate (PET) fiber exhibits remarkable thermoelectric properties, including both positive and negative Seebeck coefficients with magnitudes reaching several mV/K, along with high electrical conductivity [27]. However, while these experimental findings align with theoretical predictions in terms of performance, the underlying mechanism responsible for these unique thermoelectric properties remains unclear. Current experimental observations have only confirmed molecular distortion, leaving the electrical conduction mechanism largely unexplored. To bridge this gap between theoretical understanding and experimental observations, this study investigates the relationship between PTSA concentration and thermoelectric properties in PEDOT:PTSA materials supported on PET fibers.

## 2. Materials and Methods

The samples were prepared by polymerizing PEDOT and loading it with PTSA onto felt fabrics (100% PET) in a reaction solution. For the polymerization reaction solution, 3,4-Ethylenedioxytiophene (EDOT > 98.0%, Tokyo Chemical Industry Co., Ltd., Tokyo, Japan), Sodiumperoxyodisulfate (SPS > 97.0%, Fujifilm Wako Pure Chemicals Co., Ltd., Osaka, Japan), and p-toluenesulfonic acid (PTSA > 99.0%, Fujifilm Wako Pure Chemicals Co., Ltd.) were mixed and dispersed in purified water in the mixing ratios shown in Table 1. The felt fabrics were immersed in the polymerization reaction solution under the conditions of 296 K for 24 h with stirring at 500 rpm and then dried in a vacuum oven (DRV 220 DA, ADVANTEC, Tokyo, Japan) at 336 K.

Each sample was observed via scanning electron microscope (JCM-6000PLUS Neo scope, JEOL, Tokyo, Japan) and analyzed via a Raman scattering spectroscope (inVia Reflex, Renishaw plc., Wotton-under-Edge, UK) using a laser of a wavelength of 532 nm. The thermoelectromotive force was measured by applying a temperature difference to the front and back of the samples at a sample center temperature of 292 K using a Peltier unit via an aluminum electrode. A digital multimeter (Keithley 2100 6^1/2^ Digit Digital Multimeter, Tektronix, Inc., Beaverton, OR, USA) was employed to measure the voltage differential between the aluminum electrodes, which represents the thermoelectromotive force. Measurements were performed at 60% RH. Sheet resistivity was measured by the two-terminal method using an LCR meter (ZM2376, NF Corporation, Yokohama, Japan) in a frequency range between 1 Hz and 3 MHz. The LCR meter and sample were connected via a coaxial cable and stainless-steel electrode. Measurements were performed at 60% RH and temperatures between 293 and 323 K.

## 3. Results

### 3.1. SEM

**Figure 1:**
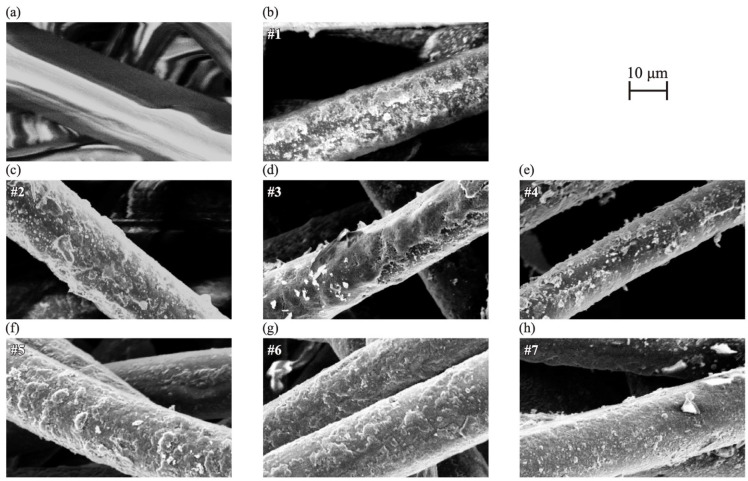
SEM micrograph of (**a**) the pristine felt fiber and (**b**–**h**) samples #1–#7.

Figure 1 shows the SEM images of each sample. In Figure 1b–h, adhesions on the fiber surface were observed, unlike in Figure 1a, which shows untreated felt. The untreated felt shown in Figure 1a was charged, suggesting that it did not exhibit conductivity. On the other hand, the samples in Figure 1b–h show clear images because of their conductive surfaces. Thus, the fiber surface was considered to be coated with a conductive material for all the PEDOT:PTSA samples. Although variations in the surface characteristics were observed among the samples, there was no trend depending on the concentration of PTSA added.

### 3.2. Raman Scattering Spectroscopy

**Figure 2:**
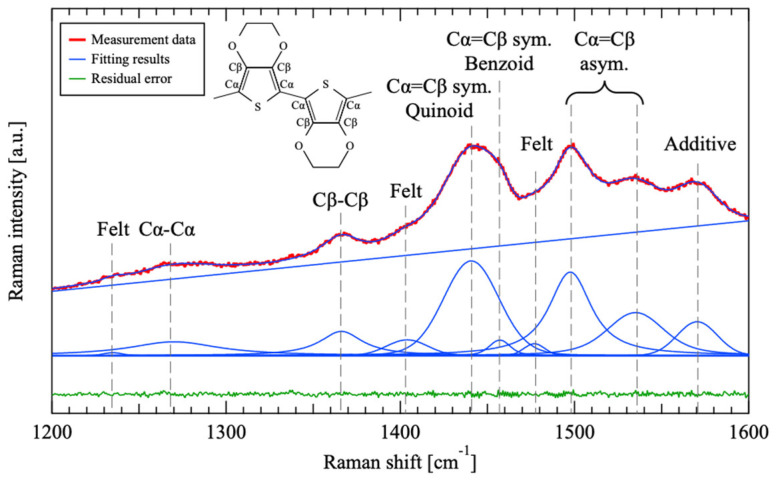
Raman spectra of sample #5 with fitting results and residual error fitted using the Voigt function.

Figure 2 shows the Raman spectra of sample #5, which has an intermediate PTSA concentration among all the samples, as a representative. In Figure 2, the blue line represents the result of the fitting, and the green line represents the residual error. A linear function was employed for the baseline during the fitting process, whereas forked functions were used for each peak. The same peak component as #5 was applied to fit all other samples, achieving a determination coefficient of roughly 99.8% to 99.9%. Peaks corresponding to the Cα-Cα, Cβ-Cβ, and Cα=Cβ bonds, which are characteristic of PEDOT [28,29], were observed in all the samples. This observation indicates successful polymerization and deposition of PEDOT. The SEM images revealed that conductive materials were deposited on the fibers across all samples. These findings suggest that a conductive material based on PEDOT was successfully deposited onto the felt fibers.

**Figure 3:**
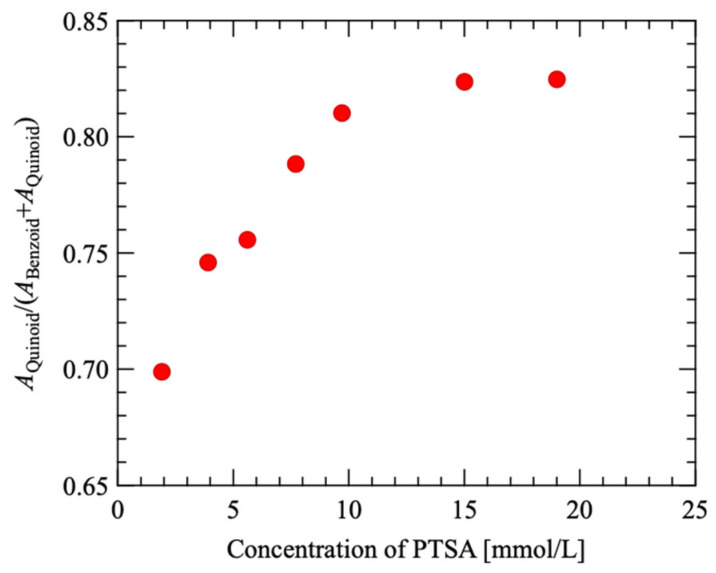
*A*_Quinoid_/(*A*_Benzoid_ + *A*_Quinoid_) as a function of PTSA concentration in polymerization solution.

PEDOT’s five-membered ring is characterized by two structural types: benzoid and quinoid. In Raman spectroscopy, peaks around 1460 cm^−1^ and 1440 cm^−1^ correspond to the benzoid and quinoid structures, respectively [29,30,31]. The ratio of the peak area of the quinoid structure (*A*_Quinoid_) to the sum of the peak areas of both structures (*A*_Benzoid_ + *A*_Quinoid_) was determined. Figure 3 illustrates the relationship between the PTSA concentration in the polymerization solution and *A*_Quinoid_/(*A*_Benzoid_ + *A*_Quinoid_). The graph demonstrates that *A*_Quinoid_/(*A*_Benzoid_ + *A*_Quinoid_) increased with increasing PTSA concentration, reaching approximately 0.82 when the PTSA concentration surpassed 10 mmol/L.

The transformation from the benzoid structure to the quinoid structure is due in part to the oxidation of PEDOT by an oxidizing agent [29,30]. Since PTSA also acts as an oxidizing agent, it is believed that, at concentrations below 10 mmol/L, increasing PTSA levels promotes PEDOT oxidation, resulting in more benzoid structures transitioning to quinoid structures. However, when the PTSA concentration exceeded 10 mmol/L, the ratio of *A*_Quinoid_/(*A*_Benzoid_ + *A*_Quinoid_) plateaued at approximately 0.82, indicating that additional PTSA did not significantly enhance PEDOT oxidation. Consequently, adding PTSA beyond 10 mmol/L to the reaction mixture was considered excessive, with a small contribution to PEDOT oxidation.

### 3.3. Thermoelectromotive Force

**Figure 4:**
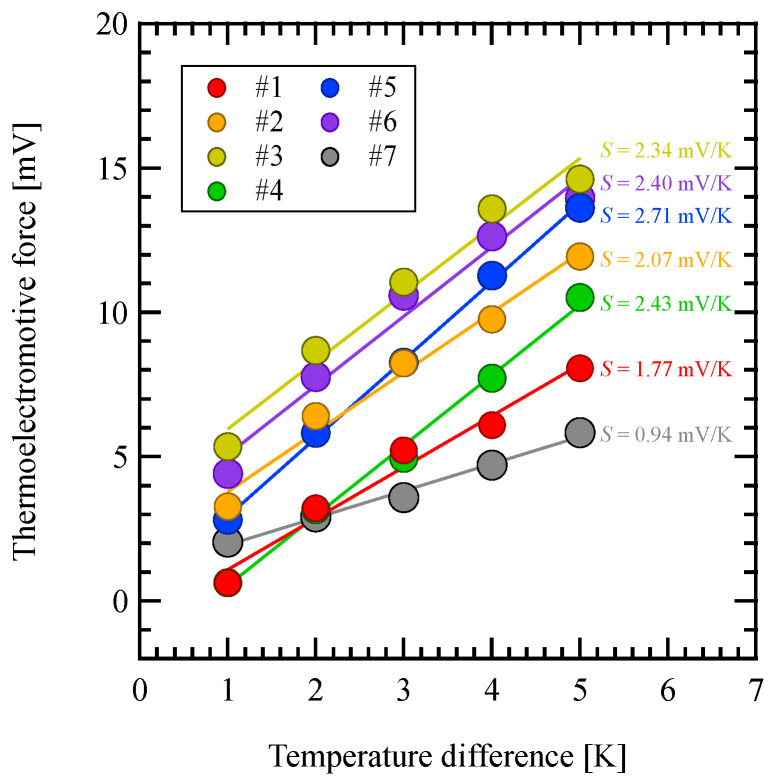
Thermoelectromotive force as a function of the temperature difference at 293 K.

Figure 4 shows the relationship between the thermoelectromotive force and temperature difference. The positive slope for all the samples indicates that they are p-type thermoelectric materials. A linear function was applied to each plot in Figure 4, and the Seebeck coefficient was derived from the resulting slope. The coefficient of determination is 97.2% to 99.8%. The slope was approximately 0.9 mV/K to 2.7 mV/K, indicating a high Seebeck coefficient.

### 3.4. Sheet Resistivity

**Figure 5:**
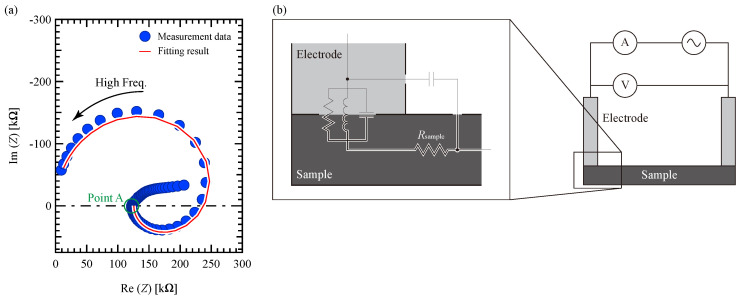
(**a**) Cole–Cole plot of sample #5 at 293 K and (**b**) equivalent circuit of the two-terminal measurement.

Figure 5a shows a Cole–Cole plot of #5 measured at 293 K as a representation. In Figure 5a, the solid line curve is obtained by fitting the equivalent circuit described below. The Cole–Cole plot exhibited two distinct regions: a curved section at higher frequencies beyond Point A and a linear portion at lower frequencies. The linear segment in the low-frequency region is typically attributed to a diffusion process occurring at the electrode interface under an alternating current (AC) electric field [32]. Because this study focuses on the electrical characteristics of thermoelectric conversion materials in relation to the charge transfer within the material under a direct current (DC) electric field, the analysis concentrates on the curved shape observed in the high-frequency region.

The carriers’ reaction to the AC electric field exhibits a semicircular pattern in the negative imaginary region, which can be modeled using an RC parallel equivalent circuit. However, the graph in Figure 5a shows a shift from a semicircle in the negative imaginary area to a positive imaginary region, accompanied by a collapse along the real axis. The semicircle in the positive imaginary region at low frequencies is discussed using an RLC parallel circuit as an equivalent circuit. The Cole–Cole plot was obtained using a two-terminal measurement technique, which incorporated the contact element between the sample and the electrode. This indicates that the two-terminal measurement method, as illustrated by the equivalent circuit in Figure 5b, consists of an RLC parallel circuit representing the contact point between the electrode and sample connected in series with the *R*_sample_ on the surface of the sample.

In RLC parallel circuits, the component described has been identified as the behavior occurring at the interface between the semiconducting material and the electrode, specifically the contact component, in solar cells [33,34,35,36]. This component is attributed to the reversal of the charge accumulation rate at the interface and the discharge rate from the interface due to the applied voltage [37]. The measurement system in this study is composed of connected electrodes and semiconducting materials, such as solar cells; thus, the equivalent circuit shown in Figure 5b is considered applicable.

Some of the Cole–Cole plots of the samples and measurement temperatures other than those shown in Figure 5a did not contain a semicircle in the positive imaginary region. However, even in these plots, the semicircles in the negative imaginary part collapsed along the real axis. Thus, the semicircles in the positive imaginary regions were not shown on the curve because the capacitance of the contact component was close to the capacitance acting on both the material-derived resistance and contact component, although the contact component described by the RLC parallel circuit was included.

**Figure 6:**
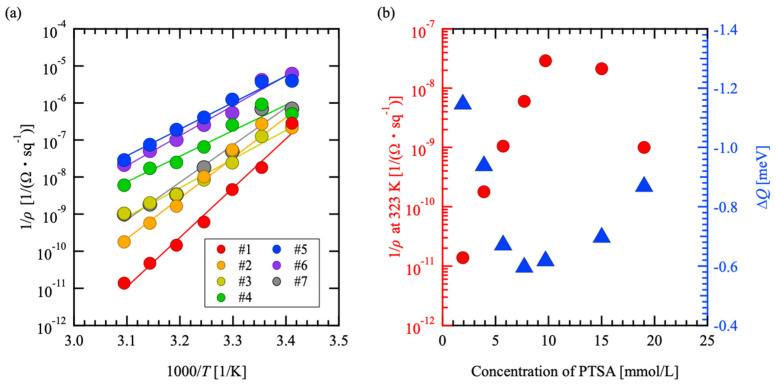
(**a**) Electrical conductivity 1/*ρ* as a function of temperature is plotted against *T*^−1^ and (**b**) PTSA concentration dependence of electrical conductivity 1/*ρ* at 323 K and Δ*Q*.

The DC resistance *R*_sample_ of the sample surface in Figure 5b was calculated by fitting the circular shape of the Cole–Cole plot using the equivalent circuit shown in Figure 5b. The resistivity calculated from *R*_sample_ was defined as the sheet resistivity *ρ*. Figure 6a shows the temperature dependence of the inverse of the sheet resistivity *ρ* corresponding to the electrical conductivity as an Arrhenius plot. The Arrhenius plots for all samples show that the conductivity decreases with increasing temperature. This is a metallic tendency; thus, it indicates that the electrical conduction mechanism is not ionic or hopping conduction.

The slope of the Arrhenius plot in Figure 6a was multiplied by -*k*_B_ and taken as the activation energy Δ*Q*. Figure 6b shows 1/*ρ* and Δ*Q* at 323 K as a function of the PTSA concentration. Both 1/*ρ* and Δ*Q* have extreme values for the PTSA concentration. The PTSA concentrations near the extremes of 1/*ρ* and Δ*Q* were consistent with the saturation of the quinoid structure transformation within the PEDOT chain, as shown in Figure 3.

The transition from the benzoid structure to the quinoid structure is due to the oxidation of PEDOT, which is also known as chemical doping. The quinoid structure is known to have high mobility owing to its linear configuration, which results from the strong PEDOT chain interaction in comparison with the benzoid structure [29,30,31]. Thus, the transformation to the quinoid structure is thought to have reduced the charge localization, resulting in a decrease in |Δ*Q*|.

At the PTSA concentration range in which the transition to the quinoid structure saturates in Figure 3, |Δ*Q*| increases with an increasing PTSA concentration. This suggests that excess PTSA increases carrier scattering without chemical doping. It has been reported that excess oxidants remain in PEDOT-based materials that have been synthesized in a reaction field [38,39] in the same way as in this study, suggesting that PTSA, which does not contribute to chemical doping in the PEDOT matrix, remains. The added PTSA may have locally and unevenly distorted the PEDOT chains to the extent that it slightly broadened the Raman spectrum. Therefore, the dependence of Δ*Q* on PTSA concentration is thought to have an extreme value owing to a decrease caused by chemical doping and an increase caused by scattering that does not involve chemical doping.

Chemical doping increases the carrier concentration in addition to weakening charge localization. In other words, the 1/*ρ* dependence on PTSA concentration is thought to have an extreme value because of the increase in carrier concentration with increasing PTSA concentration, in addition to the PTSA concentration’s dependence on Δ*Q*, until the chemical doping saturates. In Figure 6b, the slight difference in the PTSA concentration that causes the extreme values for 1/*ρ* and Δ*Q* is thought to be because the PTSA concentration that cancels out the decrease in |Δ*Q*| due to chemical doping and the increase in |Δ*Q*| due to scattering without chemical doping are lower than the PTSA concentration, causing the carrier concentration to saturate because of chemical doping. Therefore, the added PTSA contributes not only to Δ*Q* but also to the increase in carrier concentration due to chemical doping with regard to the electrical conductivity of the PEDOT:PTSA thermoelectric conversion material supported on the PET fiber.

First-principles calculations using the models of PEDOT with PTSA addition failed to show any tendency for electrical conductivity changes as PTSA concentration increased [26]. This result is explained by the fact that the calculations did not consider PEDOT’s structural transformation and defective structure derived from felt. It is suggested that enhancing the precision of theoretical analysis could strengthen the discussion on the relationship between PEDOT’s structural changes and scattering and its electrical conductivity. To achieve this, the recently proposed finite element method (FEM)-based analysis [40] and molecular dynamics (MD) method are considered potentially effective approaches.

The PEDOT:PTSA supported on PET fiber exhibited a Seebeck coefficient of several mV/K, which is approximately 10 times greater than the 150 μV/K [41] reported for Bi_2_Te_3_ and the 111 μV/K [42] observed in other PEDOT-based thermoelectric material. This significant increase in the Seebeck coefficient has a substantial impact on the conversion efficiency because it contributes to the square of both ZT and PF, resulting in a 100-fold effect. Additionally, the electrical conductivity was found to increase by several orders of magnitude, depending on the concentration of added PTSA. This suggests that PEDOT:PTSA deposited on PET fibers is promising for practical applications. However, to conduct a more precise analysis, it is essential to develop a model that accounts for the random structure of felt, enabling accurate calculations of volume conductivity in future studies. Beyond its physical attributes, this material is a conductive polymer that offers excellent processability and cost-effective production, rendering it highly valuable for flexible thermoelectric modules.

## 4. Conclusions

This study has elucidated the complex relationship between PTSA concentration and the electrical conduction mechanism in PEDOT:PTSA thermoelectric materials supported on PET fiber. Through Raman spectroscopic analysis, we demonstrated that PTSA addition systematically transforms PEDOT’s molecular structure from a benzoid to a quinoid configuration, with this transformation saturating in higher PTSA concentrations. The electrical characterization revealed two distinct regimes of PTSA’s influence. In the first regime, where structural transformation is active, increasing PTSA concentration enhances electrical conductivity through chemical doping, simultaneously reducing activation energy and increasing carrier concentration. This improvement is attributed to the quinoid structure’s enhanced carrier mobility resulting from stronger PEDOT chain interactions. However, in the second regime, where structural transformation has saturated, excess PTSA acts as a scattering center, increasing activation energy and reducing electrical conductivity without contributing to chemical doping. This dual mechanism was further supported by the observation of extreme values in both electrical conductivity and activation energy, coinciding with the saturation point of quinoid structure formation. These findings provide crucial insights into optimizing PTSA concentrations for achieving maximum thermoelectric performance, demonstrating that the material’s electrical properties are governed by the delicate balance between beneficial chemical doping effects and detrimental carrier scattering mechanisms.

The results of this research show that, by simply controlling the concentration of the PTSA added, it is possible to obtain a conductivity that is several orders of magnitude higher while maintaining a high thermoelectric power of several mV/K. This has two advantages for use in IoT devices and other applications. One is that it makes it easier to increase the voltage using an inverter, and the other is that it reduces the internal resistance of the device. These improvements contribute greatly to the practical application of the technology.

## Figures and Tables

**Table 1:** Molar densities of monomers and additives in the reaction solution.

Sample Number	EDOT [μmol/L]	SPS [mmol/L]	PTSA [mmol/L]
#1	12	14	1.9
#2	12	14	3.9
#3	12	14	5.6
#4	12	14	7.7
#5	12	14	9.7
#6	12	14	15
#7	12	14	19

## Data Availability

The original contributions presented in this study are included in the article. Further inquiries can be directed to the corresponding author.
